# The landscape of cellular immune alteration in systemic lupus erythematosus

**DOI:** 10.3389/fimmu.2026.1755310

**Published:** 2026-05-29

**Authors:** Jie Xiao, Lina Duan, Jia Yang, Yulu Deng, Shuyin Pang, Hongxia Wang, Xiaofeng Yin, Haifang Wang, Yurong Qiu, Xin Li, Ying Gong, Haixia Li

**Affiliations:** 1 Department of Laboratory Medicine, Guangdong Provincial Key Laboratory of Precision Medical Diagnostics, Guangdong Engineering and Technology Research Center for Rapid Diagnostic Biosensors, Guangdong Provincial Key Laboratory of Single-cell and Extracellular Vesicles, Nanfang Hospital, Southern Medical University, Guangzhou, China; 2 Guangdong Provincial Clinical Research Center for Laboratory Medicine , Guangzhou, China; 3 Huayin Medical Laboratory Center Co., Ltd, Guangzhou, China; 4 Division of Hematology, Department of Internal Medicine, Maastricht University Medical Center+, Maastricht, Netherlands

**Keywords:** autoantibodies, autoantigen, cellular immune, gut microbiome, immune equilibrium, systemic lupus erythematosus

## Abstract

Systemic lupus erythematosus (SLE) is a chronic autoimmune disease characterized by multi-organ inflammation and profound immune dysregulation. Aberrant interactions among adaptive and innate immune cells—including T cells, B cells, dendritic cells, macrophages, neutrophils, and natural killer cells—disrupt immune tolerance and perpetuate chronic inflammation. This review provides a comprehensive overview of the dysfunctional cellular immune landscape in SLE, focusing on the pathogenic crosstalk among immune cell subsets and its contribution to disease progression. We highlight the imbalance of T cell subsets (Th1, Th17, Tfh, Treg), B cell hyperactivation, and impaired regulatory cell function. Furthermore, we discuss how excessive NETosis, type I interferon signaling, and impaired apoptotic clearance amplify autoantibody production and immune complex-mediated injury. Emerging evidence positions gut microbiome dysbiosis as a critical environmental driver of immune dysregulation in SLE, characterized by depletion of beneficial butyrate-producing commensals and enrichment of pro-inflammatory taxa. This dysbiosis contributes to disease pathogenesis through gut barrier dysfunction, molecular mimicry, and short-chain fatty acid deficiency. Finally, we examine potential therapeutic strategies, including immune checkpoint modulation, metabolic interventions, and novel cellular therapies, aimed at restoring immune equilibrium in SLE.

## 1 Introduction

Systemic lupus erythematosus (SLE) is a chronic autoimmune disorder characterized by loss of immune tolerance and persistent multi-organ inflammation. Hallmarks include production of autoantibodies against nuclear antigens, immune-complex deposition, and resulting tissue inflammation—most notably in the skin, joints and kidneys. Clinically, SLE manifests across cutaneous, mucosal, musculoskeletal, hematologic and renal systems, with symptoms ranging from fever, hair loss and mucocutaneous lesions to life-threatening lupus nephritis (1). Globally, SLE affects an estimated 3.4 million individuals, with approximately 90% of cases occurring in women. The highest incidence is observed among Black women, reaching 230.9 cases per 100,000 person-years (1, 2). The pathogenesis of SLE is complex, involving the combined effects of multiple factors such as genetics, environment, epigenetics, and immune regulation (3).

Dysregulation of both adaptive and innate immunity lies at the core of SLE pathogenesis (4, 5). T cells exhibit aberrant activation, altered subset balance (Th17/Tfh expansion with Treg impairment), often accompanied by enhanced glycolysis and mTOR activation (2, 6, 7). B cells contribute through autoantibody production, antigen presentation, and cytokine secretion, with disrupted tolerance allowing autoreactive clones to survive and expand (8, 9). Among innate effectors, plasmacytoid dendritic cells (pDCs) overproduce type I interferon (IFN-α) via TLR7/9 signaling, amplifying inflammation and autoreactive lymphocyte activation (10, 11). Conventional dendritic cells (cDCs) upregulate co-stimulatory molecules (CD80/CD86), further breaking immune tolerance (12). Defective clearance of apoptotic debris by dendritic cells leads to persistent nuclear antigen exposure and autoantibody production (13). Abnormal interactions between pDCs and follicular helper T cells (Tfh) promote germinal center reactions and tissue damage (14). Macrophages shift toward M1 polarization (CD68^+^CD80^+^), secreting TNF-α, IL-6, and IL-1β, sustaining chronic inflammation (15), while impaired efferocytosis allows accumulation of nuclear antigens and activation of autoreactive responses (16). SLE macrophages also exhibit a tendency toward glycolytic metabolism, which may support their pro-inflammatory phenotype (17, 18). In SLE kidneys, infiltrating macrophages exacerbate glomerular damage by secreting matrix-degrading enzymes (19). Neutrophils represent another critical innate effector. Neutrophils release DNA–histone complexes through excessive NETosis, exposing self-antigens and activating pDCs to produce IFN-α, forming an inflammatory feed-forward loop (20–22). The low-density granulocyte (LDG) subset is markedly increased, and its enhanced cytotoxicity and NET formation correlate positively with renal inflammation and disease activity (23). Activated SLE neutrophils exhibit enhanced NETosis and increased ROS production, which are associated with disease activity (24). Natural killer (NK) cells show impaired cytotoxicity (reduced granzyme B/perforin) and cytokine imbalance (increased IL-6, decreased IFN-γ), resulting in defective elimination of autoreactive lymphocytes (25). Expansion of the CD56^bright^ subset and upregulation of inhibitory receptors such as NKG2A further aggravate inflammation (26), while defective NK–DC interactions contribute to insufficient apoptotic cell clearance and enhanced self-antigen presentation (27). NK cell dysfunction is closely associated with immune complex deposition and progression of renal injury (28). In parallel, dysregulated regulated-cell-death pathways—such as apoptosis, necroptosis, pyroptosis, and ferroptosis—release intracellular components as self-antigens and danger signals, further promoting autoimmune responses (29, 30).

Importantly, these cellular abnormalities do not operate in isolation. Emerging evidence positions the gut microbiome as a key environmental modulator: dysbiosis—characterized by reduced short-chain fatty acid (SCFA)-producing bacteria (e.g., *Faecalibacterium prausnitzii*) and expansion of pro-inflammatory taxa (e.g., *Ruminococcus gnavus*)—contributes to Th17/Treg imbalance, B cell activation, and systemic inflammation through microbial metabolites, molecular mimicry, and gut barrier dysfunction (31–33).

Together, the disruption of adaptive and innate immune homeostasis, reinforced by microbial factors, forms a self-amplifying loop that underlies multi-organ injury in SLE. This review integrates these dimensions into a unified framework and discusses emerging therapeutic strategies targeting cellular and microbial pathways to restore immune equilibrium.

## 2 Overview of T cell dysregulation in SLE

### 2.1 Role of T cells in autoimmunity

T cells play a central pathogenic role in SLE. In patients, aberrant expression of MHC class II—particularly HLA-DR—disrupts peripheral tolerance and facilitates autoreactive T cell activation (34).These autoreactive T cells maintain effector functions despite chronic antigen exposure, leading to sustained tissue damage (35). T cell activation is initiated through TCR recognition of antigens, including self-antigens, followed by intracellular signal transduction. In SLE, the TCR–CD3 complex undergoes structural remodeling, resulting in aberrant signaling (3). Moreover, T cells actively promote inflammatory and autoimmune processes by secreting proinflammatory cytokines such as interferon-gamma (IFN-γ), interleukin-21 (IL-21), and interleukin-17 (IL-17). In lupus nephritis (LN), T cells infiltrate renal tissues and collaborate with B cells in local immune responses, potentially exacerbating renal damage. Furthermore, T cells may intensify renal inflammation by modulating the function of other immune cells, including dendritic cells (36). T cell dysfunction in SLE extends beyond cytokine secretion: SLE T cells often exhibit altered metabolic profiles that accompany their pathogenic activation (37). A hallmark of autoimmune pathology is the imbalance between Th17 and Treg cells. Pathogenic Th17 cells expand, while Tregs are numerically and functionally impaired, promoting chronic inflammation. Recent meta-analyses have confirmed that circulating Tfh cells are significantly elevated in SLE patients (SMD 0.904, [0.620, 1.188], p < 0.01), underscoring their role in driving autoantibody production and disease activity (38).The immunometabolite itaconate has shown promise in restoring Th17/Treg balance by suppressing RORγt expression via inhibition of glycolysis and oxidative phosphorylation (OXPHOS), enhancing Treg differentiation—demonstrated in models such as encephalomyelitis (EAE) (39).

The major metabolic defects contributing to SLE T cell dysfunction are outlined in **Table 1**.

**Table 1:** Metabolic dysregulation in SLE T cells.

Metabolic defect	Pathological features	Molecular basis	Clinical consequences	References
Mitochondrial dysfunction	Elevated transmembrane potential	mTOR hyperactivation	Impaired activation-induced cell death	(40)
Enhanced glycolysis	Increased lactate production	Upregulated GLUT1 transporters	T cell hyperactivation	(41)
Oxidative phosphorylation defects	ROS overproduction	NADPH depletion, glutathione exhaustion	Prolonged oxidative stress	(42)
Lipid raft reorganization	Membrane microdomain clustering	PI3K/AKT signaling dysregulation	Sustained TCR signaling	(43)

Furthermore, T cells mediate organ damage via cytotoxic cytokine secretion and induction of apoptosis. Th1 cells and CD8^+^ T cells secrete IFN-γ or engage Fas/FasL pathways to induce tissue injury (44). Beyond their direct cytotoxic functions, T cells play a pivotal regulatory role in assisting B cells in autoantibody production. Tfh cells facilitate germinal center (GC) formation and enhance the affinity maturation of autoreactive B cells via cognate interactions, thereby exacerbating the autoimmune cascade. In diseases such as SLE, T cells aid B cells in producing large quantities of autoantibodies, which form immune complexes that deposit in target organs, inciting localized inflammation and tissue damage (45). Tregs act as crucial suppressors of immune overactivation by secreting immunomodulatory cytokines such as interleukin-10 (IL-10) and transforming growth factor-beta (TGF-β), as well as by expressing the inhibitory receptor CTLA-4. In SLE, Treg function is frequently compromised or their numbers diminished, resulting in a failure to restrain autoreactive immune responses (46).

In summary, T cells contribute to autoimmunity through diverse mechanisms: cytotoxicity, B cell help, cytokine secretion, and dysregulation of immune tolerance. Their functional plasticity and pathogenic persistence make them central targets for therapeutic intervention in SLE.

### 2.2 Abnormal T cell activation and differentiation in SLE

T cell dysfunction is central to the pathogenesis of SLE, driven by interconnected abnormalities in signaling, epigenetics, and metabolic regulation. In SLE, TCR signaling is dysregulated: T cells exhibit reduced stability and increased degradation of CD3ζ, which is replaced functionally by Fc receptor γ chains (FcRγ), redirecting signaling from ZAP-70 to Syk kinase. This reconfiguration enhances calcium influx, hyperactivates NFATC2, and upregulates co-stimulatory molecules such as CD40L, lowering activation thresholds and sustaining pathogenic T–B cell interactions (47, 48). Lipid rafts, which normally aggregate transiently upon activation, are constitutively pre-clustered in SLE T cells, further amplifying TCR/CD3 signaling (49–51). Aberrant TCR signaling also associates with reduced repertoire diversity, clonal expansion of TCR-β chains, and altered TRBV/TRBJ usage, facilitating autoreactive responses (52, 53).

Recent studies have uncovered novel molecular mechanisms governing T cell fate in SLE. Ji et al. demonstrated that accumulation of cytosolic mitochondrial DNA (mtDNA) in SLE T cells is sensed by ecto-nucleotide pyrophosphatase/phosphodiesterase 1 (ENPP1), which enhances GLUT1 transcription and glycolysis. This metabolic shift impairs AMPK activation and leads to mTORC1 hyperactivation, driving aberrant effector T cell differentiation (6). Interferon regulatory factor-1 (IRF-1) has also been identified as a key mediator of T-cell immune imbalance in SLE, with single-cell analyses revealing significant downregulation of IRF-1 in T-cell subsets during active disease (54). Epigenetic regulation adds another layer of complexity. LncRNA PVT1 is significantly upregulated in SLE patients and acts as a ceRNA to sponge miR-30e-5p, modulating T-bet/GATA3/RORγt/Foxp3 expression. In MRL/lpr mouse models, PVT1 knockdown increased Th1 and Treg cells while reducing Th2 and Th17 subsets, reversing lupus phenotypes (55). Similarly, miR-452-5p has been implicated in SLE pathogenesis by targeting RBL2 and impairing Treg development, with diagnostic potential (AUC = 0.902) (56).

Together, aberrant signaling, epigenetic dysregulation, and metabolic alterations converge to sustain pathological T cell activation and differentiation in SLE, laying a molecular foundation for disease progression.

### 2.3 CD4^+^ Th cells promote the onset of SLE through immune dysregulation and metabolic abnormalities

CD4^+^ Th cells are central regulators of immune dysregulation and tissue damage in SLE. Upon antigenic stimulation by dendritic cells and other APCs, naïve CD4^+^ T cells differentiate into specialized subsets, including Th1, Th2, Th17, Tfh, and inducible regulatory T (iTreg) cells (57). In SLE, this differentiation process is skewed, resulting in an imbalance among effector and regulatory subsets. Th1 cells, defined by IFN-γ secretion, mediate proinflammatory responses. Th2 cells promote humoral immunity via IL-4, IL-5, and IL-13 (58). Th17 cells, driven by RORγt and STAT3 signaling downstream of TGF-β, IL-6, IL-21, and IL-23, are significantly expanded in SLE, contributing to inflammation and tissue damage. Simultaneously, Treg cells—normally responsible for maintaining immune tolerance through IL-10 and TGF-β secretion—are numerically and functionally impaired (59, 60). This Th17/Treg disequilibrium is a hallmark of autoimmunity, leading to persistent inflammation and immune tolerance breakdown. Additionally, expansion of Tfh cells facilitates B cell maturation and autoantibody production, further perpetuating autoimmune pathology. A recent systematic review and meta-analysis confirmed that circulating Tfh cells are significantly increased in SLE patients across different geographical regions and phenotype markers (38).

Upon activation, CD4^+^ T cells undergo metabolic changes characterized by increased glycolytic flux—a critical adaptation for the acquisition of effector function acquisition. In both SLE patients and lupus-prone murine models, CD4^+^ T cells display elevated glycolysis following activation. Notably, combinatorial treatment with metformin and 2-deoxy-D-glucose (2-DG) has been shown to re-establish metabolic equilibrium in T cells, suppress T cell activation, reduce autoantibody titers, and ameliorate renal pathology (41). Age-associated T helper (THA) cells are a newly identified CXCR3^mid^ effector memory CD4^+^ T cell subset that expands with aging and links immunosenescence to autoimmunity. THA cells exhibit a hybrid functional profile, combining cytotoxic activity with B cell helper capacity, enabling them to simultaneously promote tissue damage and enhance autoantibody production. Their differentiation is driven by the transcription factor ZEB2, and they possess restricted TCR repertoires, suggesting antigen-driven expansion. Gene expression signatures of THA cells correlate with disease activity in SLE and are responsive to calcineurin inhibitor treatment (61).

**Figure 1:**
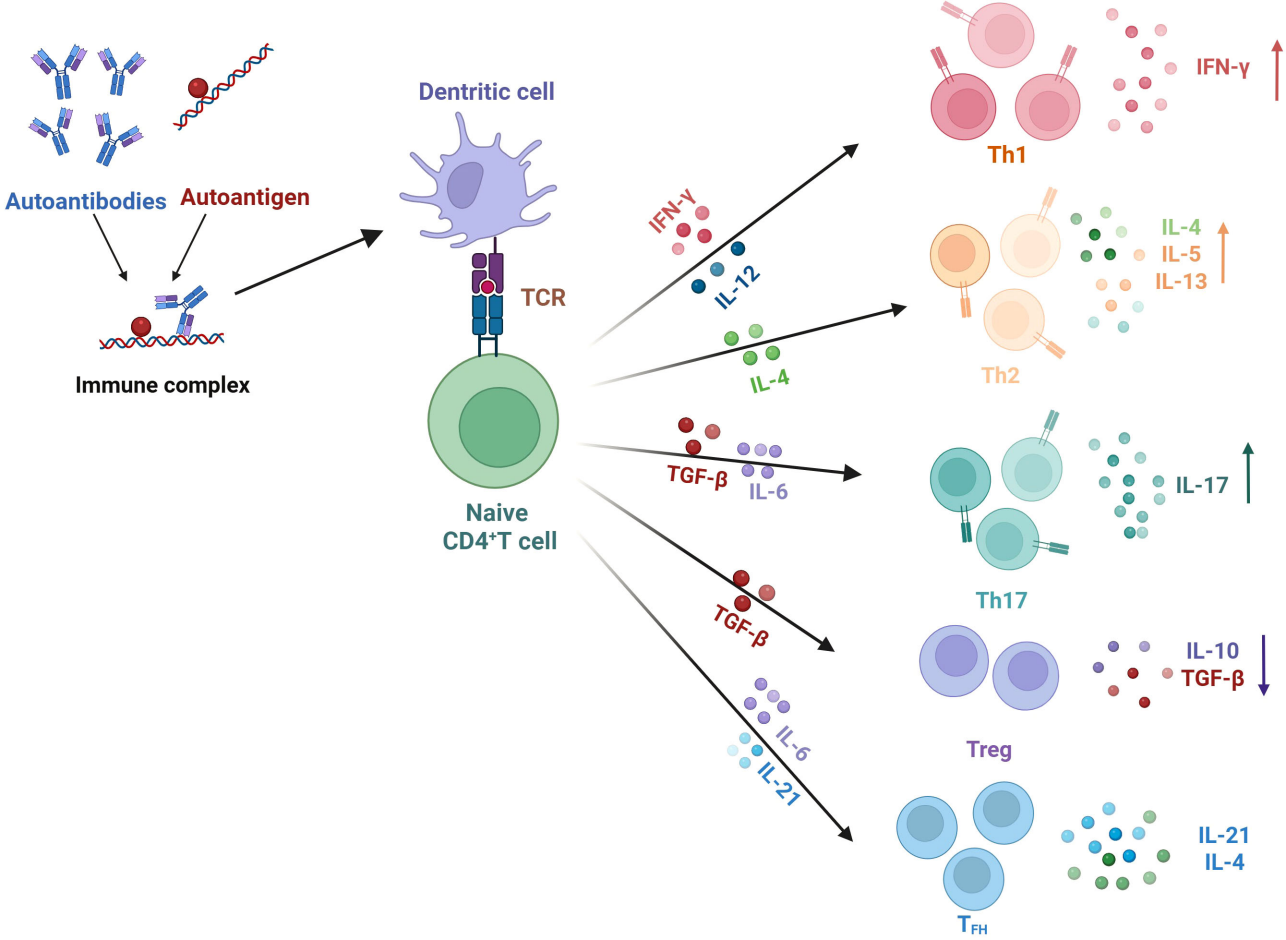
T cell dysregulation and immune imbalance in SLE. In SLE, autoantigen-autoantibody immune complexes are presented by dendritic cells to naïve CD4^+^ T cells, driving their differentiation into various T helper subsets. Cytokines such as IFN-γ, IL-12, IL-4, IL-6, TGF-β, and IL-21 guide polarization into Th1, Th2, Th17, Treg, and Tfh cells. In SLE, this process is dysregulated, leading to an increase in pathogenic Th1, Th2, Th17, and Tfh cells that secrete inflammatory cytokines (e.g., IFN-γ, IL-17, IL-21) and a reduction in functional Treg cells, weakening immune tolerance and promoting autoimmunity.

In summary, CD4^+^ T cells in SLE exhibit both functional subset imbalances and metabolic abnormalities, which together drive chronic inflammation, autoantibody production, and tissue damage (**Figure 1**).

#### 2.3.1 Th1 cell dysregulation exacerbates SLE organ damage

Th1 cells play a pathogenic role in SLE by secreting IFN-γ and synergizing with type I interferons (particularly IFN-α) to activate the STAT1 pathway. This interaction enhances the expression of proinflammatory genes, including CXCL10, and promotes a sustained immune response. IFN-γ levels are significantly elevated in SLE patients and strongly correlate with disease activity, immune complex deposition, and renal damage. It activates macrophages, induces IgG subclass switching (notably IgG2a), and contributes to tissue inflammation and mononuclear infiltration (43). Type I interferons are also upregulated in SLE, with nearly 50% of patients showing persistently elevated IFN-α levels and an even higher proportion demonstrating overexpression of IFN-α-inducible genes in peripheral blood cells. IFN-α binds to IFNARs to initiate downstream signaling, impacting multiple immune cell types—including B cells, T cells, monocytes, dendritic cells, NK cells, and neutrophils—and reinforcing autoimmune activation (62, 63). Notably, IFN-γ enhances IFN-α-induced gene expression via STAT1, while IFN-α promotes Th1 differentiation and IFN-γ production, forming a pathogenic feedback loop that amplifies systemic inflammation (62).

In SLE, IFN-α, BLyS, and IL-17 form an interconnected inflammatory axis involving T cells, B cells, neutrophils, dendritic cells, and monocytes. Increased expression of IFNAR1 and membrane-bound BLyS (mBLyS) on immune cells correlates with elevated serum IFN-α and IL-17A levels. BLyS not only supports B cell survival but also interacts with IL-17-mediated pathways, linking innate and adaptive immune responses. Notably, BLyS expression on B cells and neutrophils positively correlates with IL-17A levels, suggesting coordinated upregulation of proinflammatory pathways. *In vitro* studies further demonstrate that IFN-α directly promotes Th17 activation in SLE patients, bridging type I interferon signaling with Th17-driven pathology (64). Collectively, these findings suggest that the IFN–BLyS–IL-17 axis constitutes a self-reinforcing inflammatory network that sustains immune dysregulation and contributes to organ damage in SLE.

#### 2.3.2 Th2 cells contribute to immune alterations in SLE

Th2 cells predominantly secrete IL-4, IL-5, and IL-13, and play essential roles in humoral immunity, allergic inflammation, and certain autoimmune diseases. In SLE, Th2 cell frequency and related cytokine levels are elevated, contributing to Th1/Th2 imbalance and promoting pathogenic B cell responses (65). Th2 differentiation is primarily regulated by the IL-4/STAT6/GATA3 signaling axis, in which IL-4 induces GATA3 via STAT6 activation; GATA3 in turn enhances Th2 cytokine expression while suppressing Th1- and Th17-related genes, forming a lineage-stabilizing feedback loop (66, 67). IL-4 promotes B cell proliferation, immunoglobulin production, and class switching to IgG1 and IgE, facilitating germinal center formation, autoantibody generation, and immune complex deposition that contribute to organ damage in SLE. IL-5 recruits eosinophils into inflamed tissues such as the kidney, thereby aggravating nephritis and potentially impairing Treg function. In addition, Th2-related cytokines like IL-33 can activate pDCs, enhancing IFN-I signaling and perpetuating the inflammatory state in SLE (68–70). Basophils may further amplify Th2 polarization by responding to autoreactive IgE, enhancing memory B cell responses and promoting sustained autoantibody production (71). In short, Th2 cells contribute to immune dysregulation in SLE by driving B cell hyperactivation and reinforcing inflammatory circuits through the IL-4/STAT6/GATA3 axis and downstream interactions with pDCs and basophils.

#### 2.3.3 Molecular mechanisms of Th17 cell-mediated inflammation in SLE

Th17 cells represent a distinct subset of CD4^+^ T helper cells defined by their secretion of the proinflammatory cytokine IL-17. Their differentiation is induced by IL-6 and TGF-β, and aberrant activation can promote autoimmunity (72). In SLE, Th17 cells orchestrate inflammatory responses through IL-6/TGF-β/IL-23-dependent differentiation pathways. Elevated levels of IL-17 are observed in the peripheral blood and serum of SLE patients, alongside an increased proportion of CD4^+^ T cells producing IL-17—changes that positively correlate with disease activity (73, 74). IL-17 drives disease progression by inducing hyperproduction of IgG and anti-dsDNA antibodies in PBMCs, as well as upregulating IL-6 expression (75). Moreover, IL-17 facilitates autoantibody production by modulating chemokine activity to support GC formation and maintenance (76). The differentiation of Th17 cells is tightly regulated by cytokine networks. IL-6 and TGF-β act synergistically to initiate Th17 differentiation, with IL-6 serving as a pivotal factor in determining lineage fate—favoring Th17 development over Foxp3^+^ Treg induction. While TGF-β promotes Foxp3 expression and Treg generation, IL-6 suppresses Treg differentiation and induces IL-17 production (77). IL-23 further enhances IL-17 output by stabilizing and expanding the Th17 lineage. Notably, attenuation of lupus pathology has been reported in IL-23 receptor-deficient mice or in those treated with anti-IL-23 antibodies (78). A T cell-specific calcium/calmodulin-dependent protein kinase IV (CaMK4) enhances IL-17 gene expression through epigenetic remodeling mediated by the transcription factor CREM-α, thereby promoting Th17 differentiation. Additionally, CaMK4 activates S6K phosphorylation and facilitates RORγt nuclear translocation via the PI3K/AKT/mTOR pathway, collectively driving Th17 polarization and suppressing Treg function. The selective CaMK4 inhibitor KN-93 effectively blocks Th17 differentiation without affecting Th1 or Th2 cells, suggesting CaMK4 as a promising therapeutic target in SLE (79). Nevertheless, the contribution of Th17/IL-17A-mediated immunity appears to vary across lupus nephritis models. In MRL/lpr and NZB/NZW mice, IL-17A deficiency did not significantly impact renal histopathology or function, whereas IFN-γ blockade ameliorated disease severity (80). These findings imply that IL-17A-targeted therapies may not be universally effective across all SLE subsets.

In conclusion, Th17 cells play a crucial role in mediating inflammatory and autoimmune responses in SLE through IL-17 production.

#### 2.3.4 Regulatory T cell dysregulation drives SLE autoimmunity

Tregs are an immunosuppressive subset of T cells defined by the expression of CD4, CD25, and the transcription factor Foxp3. They play a pivotal role in preserving immune tolerance to self-antigens and restraining hyperactive immune responses (81, 82). Their functional mechanisms include direct cell contact (CTLA-4-mediated signal inhibition), secretion of inhibitory cytokines (IL-10, TGF-β, IL-35), metabolic competition (consumption of IL-2), and killing of target cells through perforin/granzyme B (GZMB) (83).

In SLE, diminished Treg cell numbers, functional impairment, and disruption of IL-2-dependent homeostasis are critical contributors to immune dysregulation (84). The reduction of circulating Tregs in SLE patients correlates with disease activity, elevated autoantibody titers, and the expansion of proinflammatory T cell subsets such as Th17 and Tfh (59). IL-2 plays a paradoxical role in SLE: it promotes effector T cell activity while supporting Treg expansion. Low-dose IL-2 therapy has emerged as a promising strategy to selectively expand Tregs and restore immune balance (85). A pronounced Th17/Treg imbalance is observed both in peripheral blood and affected tissues. Single-cell transcriptomic analysis has revealed increased infiltration of Th17 cells in the kidneys of SLE patients, correlating with urine protein/creatinine ratios. IL-17A secreted by these cells activates STAT3 signaling in renal tubular epithelial cells, inducing the release of chemokines such as CCL20 and recruiting neutrophils and monocytes, thereby establishing an inflammatory feedback loop (86). Treg suppressive capacity is primarily governed by Foxp3 expression. Reduced Foxp3 expression impairs Treg functionality, particularly their ability to restrain Th17 differentiation (87). Mechanistically, IL-2 insufficiency leads to downregulation of Foxp3, compromising Treg survival and function; IL-2 is thus indispensable for Treg homeostasis (88). In lupus mouse models (B/W and NZB×NZW F1), Treg numbers gradually decrease with disease progression, and effector T cells are overactivated, confirming that IL-2 deficiency exacerbates immune imbalance (89, 90).

Therapeutic strategies aimed at expanding Tregs or supplementing IL-2 hold promise. Low-dose recombinant human IL-2 (rhIL-2) selectively promotes Treg expansion, suppresses Th17/Tfh cells, restores effector/regulatory T cell balance, and significantly ameliorates disease activity (59, 91). In animal experiments, infusion of Tregs can reduce inflammation and organ damage in lupus mice (74), suggesting that restoring Treg function or number may reestablish immune tolerance.

#### 2.3.5 Tfh cells drive the pathological mechanism of SLE autoantibodies

Tfh cells are a specialized subset of CD4^+^ T cells critical for regulating humoral immunity (92). Their primary role involves promoting GC reactions to facilitate B cell antibody class switching and affinity maturation (92, 93). Phenotypically, Tfh cells are defined by elevated expression of CXCR5, PD-1, ICOS, and the transcription factor Bcl-6 (92, 94). Through IL-21 secretion, Tfh cells regulate B cell proliferation and plasma cell differentiation (93), while the CD40L-CD40 axis enhances B cell antigen presentation (92).

In SLE, Tfh cells drive the breakdown of immune tolerance and pathogenic autoantibody generation through dysregulated expansion, aberrant IL-21/IL-6 signaling, and Bcl-6-dependent GC abnormalities. Targeting the interaction between Tfh cells and B cells, particularly via blockade of key costimulatory molecules such as ICOS and CD40L, represents a promising therapeutic strategy. Tfh cells promote the differentiation of autoreactive B cells into plasma cells that secrete pathogenic autoantibodies (e.g., anti-dsDNA and anti-Sm antibodies) through sustained activation and aberrant interactions with B cells. This process is mediated by Tfh-derived cytokines IL-21 and IL-6, costimulatory signaling via ICOS/CD40L, upregulation of Bcl-6, and concurrent dysregulation of Treg function. In lupus-prone murine models such as MRL/lpr and NZB/NZW, Tfh cell expansion and GC dysregulation are evident, and genetic deletion of Bcl-6 or pharmacologic blockade of IL-21 ameliorates disease manifestations (45, 59, 72, 95).

### 2.4 CD8^+^T cell dysfunction promotes the development of SLE

In SLE, CD8^+^ T cells display diminished cytotoxicity and impaired antiviral defense, partly due to downregulation of SLAMF4 expression and dysfunction of CD38^+^ subpopulations. CD8^+^ T cells from SLE patients show marked SLAMF4 downregulation, resulting in a contraction of the SLAMF4^+^ CD8^+^ T cell pool. These SLAMF4^-^ CD8^+^ T cells exhibit reduced cytotoxic potential and diminished responsiveness to viral peptides. Moreover, the decline of SLAMF4^+^ memory CD8^+^ T cells is associated with loss of CD8 expression and the emergence of double-negative T cells, which may underlie impaired cytotoxic responses and elevated infection rates in SLE (96). Recent single-cell RNA and TCR sequencing studies have established a critical link between clonal expansion of effector CD8^+^ T cells and disease exacerbation in SLE. Paek et al. demonstrated that specific CD8^+^ T cell clonotypes expand during flare states, transitioning to effector phenotypes with heightened cytotoxicity and amplified interferon signaling, strongly correlating with tissue damage and flare severity (97).

Further characterization has revealed a distinct CD8^+^CD38^high^ subpopulation in SLE patients, which correlates with increased infection susceptibility. Compared to healthy individuals, CD8^+^CD38^high^ T cells exhibit defective cytotoxic activity, evidenced by impaired degranulation and reduced expression of cytolytic effectors such as granzymes A and B (GZMA, GZMB) and perforin (PRF1). These cells also show diminished expression of transcription factors critical for cytotoxic programming, including T-bet, RUNX3, and EOMES. CD38 overexpression is strongly linked to CD8^+^ T cell dysfunction and infection risk. Pharmacologic modulation of the CD38/NAD^+^/Sirtuin1/EZH2 axis offers a promising therapeutic avenue to restore CD8^+^ T cell cytotoxicity and mitigate infection in SLE (98). CTLs exert cytotoxicity by targeting self-antigen-expressing cells through the perforin-granzyme axis. In SLE-associated cutaneous lesions, CXCR3^+^ CD8^+^ T cells are recruited to the epidermis via CXCL10 secreted by keratinocytes, where they exhibit elevated granzyme B expression, leading to keratinocyte apoptosis and erythema. Renal involvement is marked by CD8^+^ T cell infiltration, which correlates with the severity of tubulointerstitial fibrosis. CTL subsets with reduced TIM-3 expression display heightened profibrotic activity (99–102). Overall, CD8^+^ T cells—though defined by their cytolytic function—frequently exhibit dysfunction in SLE, contributing to both impaired host defense and immune-mediated tissue injury (103).

Key aberrant T cell signaling pathways implicated in SLE pathogenesis are summarized in **Table 2**.

**Table 2:** Abnormal T cell signaling pathways in SLE.

Signaling pathway	Dysregulation	Key targets	Functional impact	References
TCR-CD3 complex	ζ-chain hypophosphorylation	Reduced ZAP70 activity	Lower activation threshold	(3)
CaMKIV pathway	Nuclear CaMKIV overactivation	CREMα hyperactivation	IL-2 suppression, IL-17 promotion	(3)
mTOR pathway	mTORC1/2 imbalance	Abnormal S6K phosphorylation	Th17 polarization, Treg inhibition	(104)
JAK-STAT pathway	Persistent STAT1/3 activation	Enhanced IFNAR signaling	Type I interferon signature	(62)

The roles of T cell subsets in mediating organ-specific damage in SLE are listed in **Table 3**.

**Table 3:** T cell-mediated organ damage in SLE.

Target organ	Effector subsets	Injury mechanisms	Biomarkers	References
Kidney	Th17, Tfh	IL-17-driven neutrophil infiltration	Elevated urinary IL-17	(105)
Skin	CD4^+^ memory T cells	IFN-γ-induced keratinocyte apoptosis	Cutaneous IFN signature genes	(106)
Joints	γδ T cells	RANKL-mediated osteoclast activation	Synovial IL-17A levels	(107)
Nervous system	DN T cells	Blood-brain barrier disruption	Increased CSF CXCL10	(107)

## 3 B cell dysregulation and autoantibody-driven immunopathogenesis in SLE

### 3.1 Function of B cells in the pathogenesis of SLE

B lymphocytes play a central role in SLE pathogenesis through autoantibody production, antigen presentation, and cytokine secretion. In SLE, B cell tolerance is profoundly compromised, permitting the survival, activation, and differentiation of autoreactive B cells. These cells produce autoantibodies, particularly targeting nuclear constituents such as DNA, which subsequently form immune complexes that deposit in tissues, activate the complement system, and initiate inflammatory cascades, culminating in chronic inflammation and organ damage (9, 108–110). In addition, aberrant B cell signaling leads to B cell hyperactivity and survival, influenced by overexpression of B cell activating factor (BAFF) and abnormal interactions with Tfh cells (111, 112).

Recent studies have uncovered novel epigenetic mechanisms governing B cell fate in SLE. Yang et al. demonstrated that METTL3, an m6A methyltransferase, is significantly elevated in SLE B cells and positively correlates with disease activity. Mechanistically, METTL3 binds to PAX5 mRNA, stabilizing it via m6A modification and promoting PAX5 expression; in turn, PAX5 directly binds to the METTL3 promoter, driving its expression. This reciprocal METTL3-PAX5 regulation maintains B-cell identity and promotes B-cell hyperreactivity in SLE (113).

### 3.2 Impaired B cell tolerance in SLE

In SLE, defects in these tolerance processes allow autoreactive B cells to escape elimination, survive, and activate. These defects are exacerbated by genetic and molecular abnormalities, including enhanced expression of co-stimulatory molecules such as CD40 and dysregulated B cell receptor (BCR) signaling pathways (114, 115). Genetic polymorphisms affecting key signaling molecules, including Lyn kinases and phosphatases, result in overreactive BCR signaling thresholds. This dysregulation leads to uncontrolled activation and survival of autoreactive B cells, further exacerbating the autoimmune response (116).

Epigenetic regulation plays a critical role in B cell tolerance breakdown. Jing et al. demonstrated that Methyl-CpG-binding domain protein 2 (MBD2) is highly expressed in B cells of SLE patients and positively correlates with disease activity. Mechanistically, MBD2 selectively binds to methylated CpG of Lef-1 induced by IFN-α, repressing Pten transcription and thereby promoting PI3K-Akt-mTOR signaling, which drives B cell differentiation and BCR signaling (117).

### 3.3 GC dysregulation and autoantibody production

GCs are essential for B cell maturation, somatic hypermutation, and class switch recombination, processes that are critical for antibody affinity maturation. In SLE, GCs are abnormally expanded and dysregulated. Enhanced interactions with Tfh, mediated by cytokines such as IL-21 and surface molecules such as ICOS, lead to hyperactivation of B cells within GCs. This leads to the production of high-affinity autoantibodies, including anti-double-stranded DNA (anti-dsDNA), antinuclear antibodies (ANA), and anti-Sm antibodies, which are hallmarks of SLE (118–121).

Beyond classical GCs, extrafollicular (EF) B cell responses have emerged as a major source of pathogenic autoantibodies in SLE. Wu et al. (2024) performed single-cell RNA/BCR/TCR sequencing on kidney and paired peripheral blood from patients with active lupus nephritis and identified high infiltrations of intrarenal atypical B cells (ABCs) and antibody-secreting cells (ASCs). The single-cell BCR repertoire analysis revealed strong clonal expansion of intrarenal ASCs dominated by IGHG1 and IGHG3 isotypes (122). Extrafollicular CD19^low-^CXCR5^-^CD11c^-^ double negative 3 (DN3) B cells are significantly associated with disease activity in female SLE patients, and CD19 expression level on DN cells serves as an accurate disease activity biomarker (123). Furthermore, Dang et al. identified a substantial expansion of autoreactive CD19^-^ plasma cells, particularly class-switched CXCR3^+^ and phosphatidylcholine-specific B-1-derived subsets, in lupus-prone mice. Peripheral blood from SLE patients showed elevated frequencies of CD19^-^ PCs, implicating these cells in sustaining pathogenic activity beyond CD19-targeting therapies (124).

### 3.4 Role of Bregs in SLE

Regulatory B cells (Bregs) are key modulators of immune responses in SLE. Bregs exert immunosuppressive effects primarily through IL-10 production, but can also regulate immunity via TGF-β secretion, induction of Tregs, and apoptosis of effector T cells. Their function depends on signaling molecules such as CD40, CD19, CD1d, and BCR, and defects in these pathways are associated with impaired Breg activity and enhanced proinflammatory responses (125). In SLE patients, CD19^+^CD24^hi^CD38^hi^ Bregs show functional impairment: they are resistant to CD40 stimulation, produce less IL-10, and fail to suppress Th1 cell differentiation, contributing to the breakdown of peripheral tolerance (126). A subset of IL-10–producing B10 cells, mainly within the CD24^hi^CD27^+^ population, is detectable in SLE and other autoimmune diseases, with frequencies elevated compared to healthy controls, possibly representing a compensatory regulatory response (127). Narendra et al. employed a multiomic approach to compare aging-related changes in peripheral blood immune profiles of 287 SLE patients and 928 healthy controls. In SLE patients, aging correlated with lower expression of interferon-stimulated genes (ISGs) across multiple cell types including B cells, decreased plasma IFN-α2, and differential genome methylation. Of the genes both down-regulated and hypermethylated with older age, ISGs were disproportionately represented, suggesting a role for epigenetic silencing in attenuating IFN signaling (128). Breg differentiation and function are tightly linked to mitochondrial metabolism and controlled reactive oxygen species (ROS) levels, with thioredoxin (Trx) being critical; in SLE, Bregs are deficient, display mitochondrial depolarization, elevated ROS, and reduced Trx^+^ B cells, whereas exogenous Trx can restore their regulatory function (129). Additionally, a potent subset of CD1d^hi^CD5^+^ Bregs, identified in mice, produces IL-10 and suppresses T cell–mediated inflammation; deficiency or dysfunction of this subset exacerbates autoimmunity, suggesting its potential relevance in SLE pathogenesis (130).

### 3.5 Type I interferon signature and B cell hyperactivity

A hallmark of SLE is the type I IFN signature, characterized by elevated levels of IFN-α, which results in B-cell hyperactivity. IFN-α facilitates the differentiation of B cells into plasmablasts and plasma cells while concurrently suppressing regulatory pathways. In concert with cytokines such as BAFF and APRIL (a proliferation-inducing ligand), IFN-α promotes the survival and activation of autoreactive B cells. Notably, increased levels of BAFF reduce the activation threshold of B cells, thereby sustaining autoimmunity (131–133). Studies have shown that overexpression of BAFF in transgenic mice induces B-cell proliferation, increased production of autoantibodies (including anti-dsDNA), increased serum immunoglobulin levels (IgM, IgA, IgE, IgG), and lupus-like renal changes due to immune complex deposition. These findings highlight the central role of BAFF in the pathogenesis of SLE (112, 134).

Recent clinical trials have demonstrated the efficacy of targeting the type I IFN pathway. Tang et al. reviewed the clinical pharmacokinetics, pharmacodynamics, and immunogenicity of anifrolumab, a monoclonal antibody targeting IFNAR1. The approved dosing regimen (300 mg intravenous every 4 weeks) was based on Phase 2b MUSE and Phase 3 TULIP-1/TULIP-2 trials, in which anifrolumab treatment was associated with clinically meaningful improvements in disease activity with an acceptable safety profile (135). Furthermore, A physiology-based pharmacokinetic model by Sharma et al. predicted high unbound local concentrations of anifrolumab in blood, skin, gastrointestinal tract, lungs, and muscle, exceeding its IFNAR1 dissociation equilibrium constant values, with high IFNAR1 occupancy predicted for both subcutaneous and intravenous dosing (136).

### 3.6 Synergistic effects of T cells and B cells in SLE

**Figure 2:**
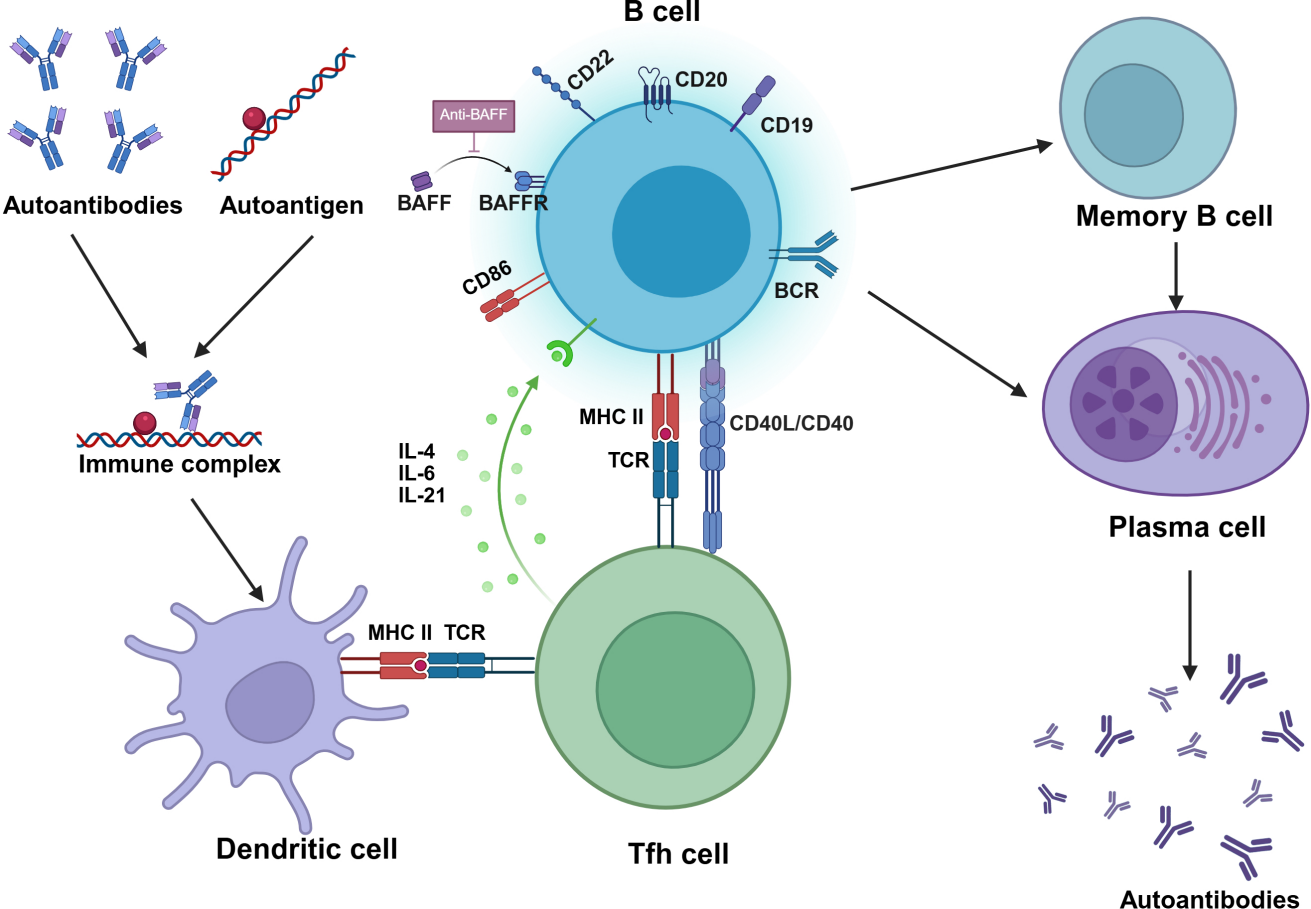
Aberrant B cell activation and autoantibody production in SLE. In SLE, the formation of immune complexes composed of autoantigens and autoantibodies activates dendritic cells, which present antigens to Tfh cells via MHC II molecules. Activated Tfh cells interact with B cells through CD40–CD40L signaling and secrete cytokines such as IL-4, IL-6, and IL-21 to promote B cell activation, proliferation, and differentiation. Dysregulated B cell activation is further supported by the BAFF–BAFFR signaling pathway, enhancing B cell survival and contributing to the expansion of autoreactive B cells. These activated B cells differentiate into memory B cells and antibody-producing plasma cells, leading to the continuous production of pathogenic autoantibodies. This autoantibody-mediated immune dysregulation is central to SLE pathogenesis, resulting in widespread tissue damage and chronic inflammation.

In SLE, T and B cells form a pathological positive feedback loop that drives chronic autoimmunity (**Figure 2**). Through direct cellular contact, T cells engage B cells via the CD40–CD40L/IL-21 signaling axis, promoting B-cell proliferation, class-switch recombination, and differentiation into plasma cells, ultimately resulting in high-affinity pathogenic anti-dsDNA antibodies and immune complex deposition (137–139). These immune complexes accumulate in target organs—such as the skin, vasculature, lungs, and kidneys—triggering complement activation and progressive tissue injury (140). Meanwhile, T cells secrete pro-survival mediators including IL-6 and BAFF, further enhancing B-cell maturation and antibody production (141). Functionally, B cells reciprocally present self-antigens to T cells via MHC class II, perpetuating autoreactive T-cell expansion and reinforcing the autoimmune cycle (43, 142). Under physiological conditions, Tregs and IL-10-producing Bregs suppress excessive immune activation; however, in SLE, these regulatory networks are disrupted, leading to unchecked T–B cell cooperation (143).

Recent evidence further reveals that this pathogenic loop is exacerbated by the abnormal expansion of CXCL13^+^PD-1^+^ICOS^+^ peripheral helper T (Tph) cells, which provide potent B-cell help outside germinal centers. Tph cells actively drive plasmablast differentiation and antibody production, and their frequencies positively correlate with overall disease activity and the severity of lupus nephritis (LN). Mechanistically, transcriptional regulation of CXCL13, a key B-cell chemoattractant, governs Tph differentiation. The aryl hydrocarbon receptor (AHR) acts as a negative regulator by cooperating with AP-1 family member JUN to inhibit CXCL13 expression and suppress Tph/Tfh-like programs, while promoting an IL-22-producing Th22 phenotype. Conversely, type I interferon, a central pathogenic mediator in SLE, antagonizes AHR-JUN signaling and promotes CXCL13 expression, driving Tph amplification (144, 145). In addition, TGF-β3 has been identified as a major inducer of Tph cells in SLE, generating programmed cell death protein 1 (PD-1)^hi^ musculoaponeurotic fibrosarcoma (MAF)^+^, IL-21^+^IL-10^+^ Tph-like cells and upregulating Tph signature genes (PDCD1, MAF, SOX4, CXCL13). These cells strongly induce class-switch memory B cells to differentiate into plasma cells and enhance antibody production, further linking Tph biology to autoreactive humoral responses (146). Clinically, the frequency of TIGIT ± PD-1^+^ Tph cells correlates positively with plasmablast expansion, reinforcing their role as accelerators of autoantibody generation (144).

Genetic and epigenetic alterations further intensify this pathogenic synergy. Polymorphisms in genes encoding IL-21 or CD40 increase susceptibility to excessive T–B cell activation (147), while DNA hypomethylation in T and B cells amplifies cellular activity and responsiveness to autoantigens (148). Given these mechanistic insights, targeted therapies that block CD40–CD40L or IL-21 signaling have shown promise in reducing pathological T–B cell collaboration (149). Moreover, restoration of Treg/Breg function may represent a crucial strategy for interrupting the self-reinforcing autoimmune circuit in SLE (143). These findings underscore the fundamental role of T–B cell synergy in disease pathogenesis and offer a conceptual foundation for the development of precision therapies targeting immune cell interactions.

The molecular interactions between T and B cells that facilitate pathogenic autoantibody production are detailed in **Table 4**.

**Table 4:** T-B cell collaborative mechanisms in SLE.

Interaction mode	Molecular basis	Pathological effects	Therapeutic targets	References
CD40-CD40L	Costimulatory signal amplification	Autoantibody class switching	CD40L monoclonal antibodies	(137)
IL-21 signaling	Tfh-B cell paracrine signaling	Pathological GC formation	IL-21 receptor antagonists	(138)
MHC-II presentation	Autoantigen presentation	Autoreactive T cell activation	HLA-DR blockers	(142)
BAFF/APRIL pathway	Enhanced B cell survival	Long-lived plasma cell generation	Belimumab (BAFF inhibitor)	(141)

## 4 Dendritic cell-mediated autoantigen presentation in SLE pathogenesis

Dendritic cells (DCs) are professional APCs that serve as a critical interface between innate and adaptive immunity by capturing, processing, and presenting antigens to naïve T cells (150). In healthy individuals, DCs maintain peripheral tolerance by inducing Tregs and limiting the activation of autoreactive lymphocytes, but in SLE, their tolerance function is lost (13, 151). Such dysfunction is characterized by impaired clearance of apoptotic cells, resulting in aberrant presentation of self-antigens, including nuclear and cytoplasmic constituents, thereby perpetuating chronic immune activation (152). The principal DC subsets—pDCs and cDCs—assume distinct pathogenic roles in SLE. Recent single-cell RNA sequencing analysis of peripheral blood mononuclear cells from SLE patients has confirmed that elevated ISG expression is largely attributable to pDCs and monocytes, with pDCs showing heightened activity of NF-κB, STAT1, STAT3, and IRF9 regulons (153, 154). Type I interferon production by pDCs was initially low in stable patients but surged during active flare, accompanied by upregulation of chemokine receptors (CCR2, CX3CR1) and activation markers, with pDCs differentiating into monocyte-like cells associated with disease exacerbation and tissue damage (155). pDCs constitute the primary source of type I interferons, particularly IFN-α, which are excessively produced upon sensing nucleic acid-containing immune complexes via TLR7/TLR9 signaling (11, 156, 157). These interferons establish a self-reinforcing loop that sustains DC activation and systemic inflammation by enhancing the activation and persistence of autoreactive T and B cells, fostering the differentiation of monocytes into inflammatory macrophages, and inducing the overexpression of interferon-stimulated genes (ISGs), collectively known as the type I interferon signature (158). In addition, pDCs abnormally accumulate in inflamed tissues, further exacerbating tissue damage (159). On the other hand, cDCs—including cDC1 and cDC2 subsets—reduce the activation threshold of T cells by upregulating costimulatory molecules (CD80/CD86), thereby expanding autoreactive T cell populations and driving the secretion of proinflammatory cytokines and the formation of pathogenic immune complexes (12, 160). Dysregulated DCs further potentiate the inflammatory milieu through interactions with other immune cell subsets. For instance, activated pDCs and cDCs stimulate Tfh cells, which directly enhance B cell activation and autoantibody production; concurrently, DCs recruit and activate inflammatory monocytes and neutrophils, contributing to organ-specific damage (14).

## 5 Macrophage-driven immune dysregulation in SLE

**Figure 3:**
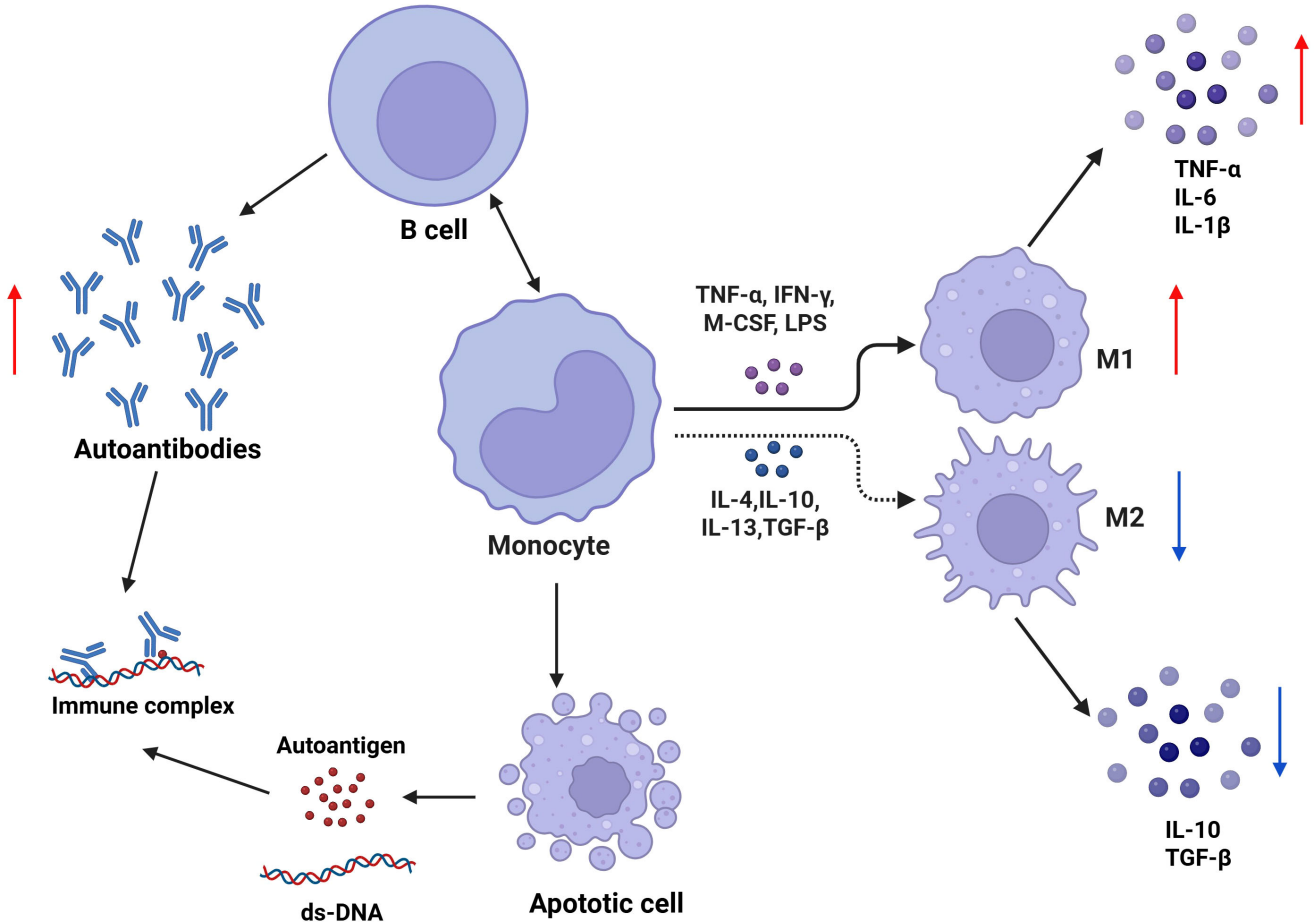
Macrophage polarization and immune dysregulation in SLE. This figure illustrates the dynamic interplay between macrophage polarization states and immune dysregulation in SLE, contrasting a balanced immune microenvironment with SLE-associated pathology. In a healthy immune system, monocytes differentiate into pro-inflammatory M1 macrophages (regulated by TNF-α, IFN-γ, and LPS) and anti-inflammatory M2 macrophages (modulated by IL-4, IL-10, IL-13, and TGF-β), maintaining homeostasis through controlled cytokine signaling (e.g., TNF-α, TGF-β) and efficient clearance of apoptotic cells to prevent autoantigen release. Conversely, in SLE, disrupted macrophage polarization favors sustained M1 dominance, exacerbating inflammation through elevated TNF-α and IFN-γ, while M2-associated cytokines (IL-10, TGF-β) fail to resolve tissue damage, leading to defective apoptotic cell clearance and accumulation of autoantigens such as ds-DNA. B cell-derived autoantibodies bind these autoantigens, forming pathogenic immune complexes that deposit in tissues, perpetuating chronic inflammation, aberrant T cell activation (Th1/Th17), and organ damage. This schematic underscores how dysregulated macrophage plasticity and cytokine networks drive immune dysfunction, linking cellular mechanisms to SLE progression and autoimmunity.

Macrophages are pivotal components of the innate immune system with remarkable plasticity. Under physiological conditions, the equilibrium between classically activated pro-inflammatory macrophages (M1, CD68^+^CD80^+^) and alternatively activated anti-inflammatory macrophages (M2, CD163^+^CD206^+^) is essential for maintaining immune homeostasis: M1 macrophages facilitate pathogen clearance via the secretion of TNF-α, IL-6, and related cytokines, whereas M2 macrophages promote tissue repair (161, 162). However, in SLE, this equilibrium is profoundly disrupted (**Figure 3**). Although the overall number of macrophages increases in peripheral blood and tissues, the proportion of M1 macrophages is abnormally elevated while M2 macrophages are diminished, resulting in excessive production of pro-inflammatory mediators (e.g., TNF-α, IL-6, IFN-γ) that drive systemic inflammation and tissue injury (19, 163, 164). The mechanisms underlying this polarization imbalance are multifactorial and intricate. Type I interferons, particularly IFN-α secreted by pDCs, induce M1-associated gene expression (e.g., iNOS, IL-12) via STAT1 phosphorylation (15, 165); the abnormal sensitivity of TLR7/9 to its own RNA/DNA complex leads to the sustained activation of the NF-κB pathway (18) the accumulation of mitochondrial reactive oxygen species (mtROS) activates the NLRP3 inflammasome and promotes the maturation of IL-1β and IL-18 (166). At the same time, the self-reinforcing secretion of IL-6 and TNF-α further exacerbates the vicious cycle of inflammation (167).

Beyond polarization abnormalities, functional impairments in SLE macrophages are also evident: their defective efferocytosis hampers the clearance of apoptotic cells, resulting in the accumulation of self-antigens and subsequent autoantibody production. This dysfunction is linked to aberrant phagocytic pathways and impaired regulation of inflammatory cytokines (16, 168). Metabolic reprogramming further contributes to this dysregulation, typified by enhanced glycolytic flux and suppressed oxidative phosphorylation (OXPHOS), favoring M1 polarization and sustaining chronic inflammation. Mitochondrial dysfunction and excessive ROS release exacerbate tissue damage (166, 169). Epigenetic dysregulation (such as hypomethylation of pro-inflammatory gene promoters and disordered miRNA expression) leads to the continuous activation of inflammatory genes (such as IL-6 and TNF-α) (118). In addition, intestinal dysbiosis also modulates macrophage function via microbial metabolites and molecular patterns. In SLE, a reduced abundance of Bacteroides—producers of polysaccharide A (PSA)—impairs IL-10 induction via TLR2 signaling, while overgrowth of Enterobacteriaceae activates the NF-κB pathway through LPS. Microbiota-derived tryptophan metabolites (e.g., kynurenine) influence macrophage polarization through the aryl hydrocarbon receptor (AhR), and imbalances in this axis are associated with cutaneous manifestations in SLE (170). Ultimately, M1-polarized macrophages infiltrate target organs such as the kidneys and skin, releasing pro-inflammatory cytokines and matrix-degrading enzymes, culminating in organ damage including lupus nephritis and cutaneous inflammation (19, 164). All in all, macrophages orchestrate immune dysregulation in SLE via M1/M2 polarization imbalance, metabolic reprogramming (enhanced glycolysis and mitochondrial dysfunction), and epigenetic abnormalities.

## 6 Neutrophil via NETosis induce pathogenesis in SLE

**Figure 4:**
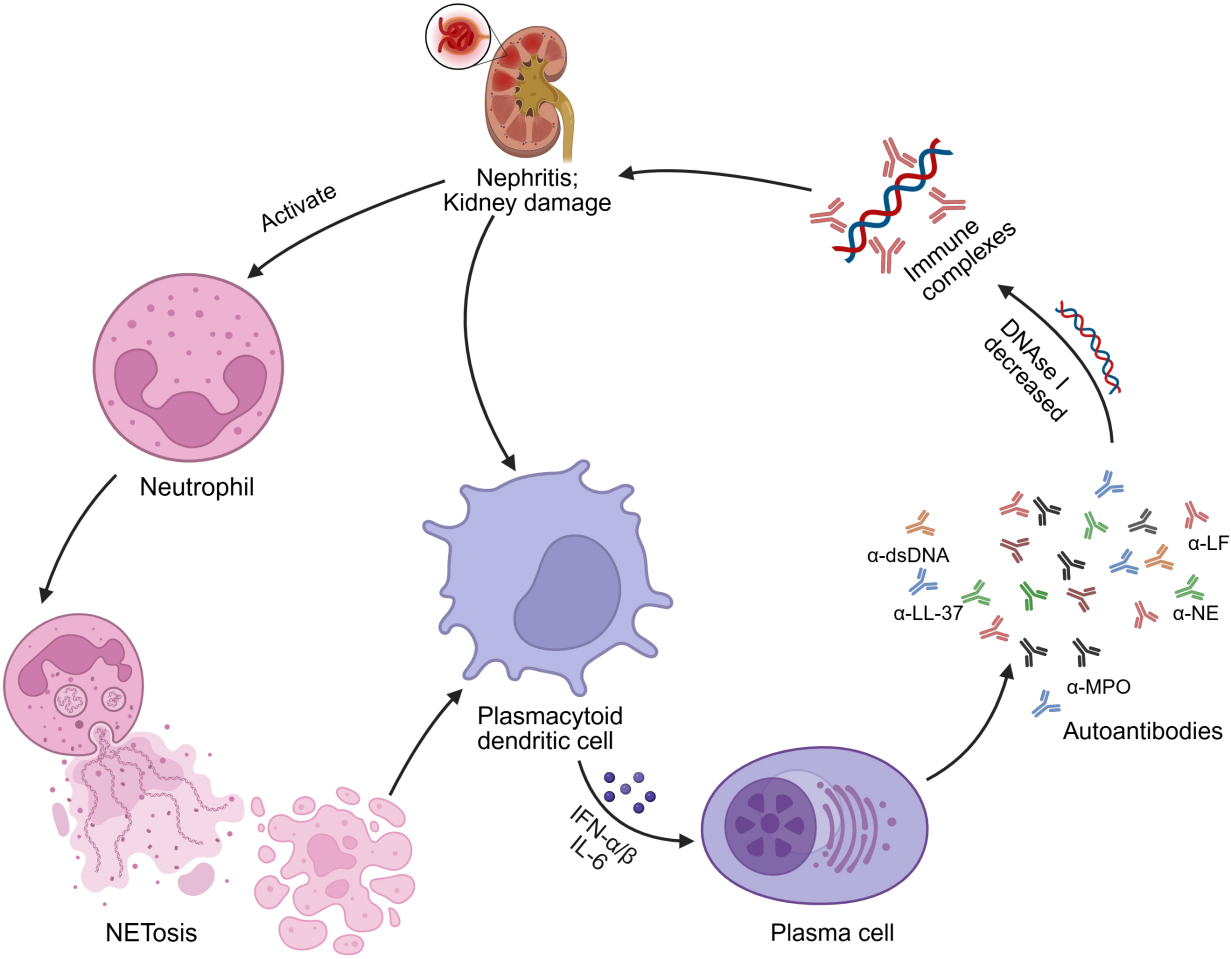
Neutrophil activation and NETosis-driven pathogenesis in SLE. This figure illustrates the critical role of neutrophils in SLE pathogenesis, highlighting their activation, NETosis (neutrophil extracellular trap formation), and contribution to immune dysregulation and organ damage, where activated neutrophils release NETs containing autoantigens such as α-lactoferrin (α-LF), LL-37, and myeloperoxidase (α-MPO), while reduced DNase I activity impairs NET degradation, leading to persistent autoantigen exposure and immune complex formation with autoantibodies targeting nuclear components (e.g., ds-DNA); these immune complexes deposit in tissues, particularly the kidneys, triggering nephritis and renal damage, and are further amplified by pDCs secreting pro-inflammatory cytokines (IFN-α/β, IL-6), which promote plasma cell differentiation and autoantibody production, while macrophages exacerbate tissue injury through phagocytosis of immune complexes and inflammatory mediator release, collectively driving the chronic inflammation and autoimmune responses characteristic of SLE.

Neutrophils are the most abundant leukocytes in circulation. In SLE, both the number and function of neutrophils are markedly dysregulated (**Figure 4**). Excessive complement activation results in premature depletion of circulating neutrophils, causing neutropenia, while the population of LDGs—a proinflammatory neutrophil subset—increases significantly. LDGs exhibit heightened cytotoxicity, induce vascular injury and endothelial dysfunction, and demonstrate an enhanced capacity for neutrophil extracellular trap (NET) formation. Their transcriptional profile is strongly associated with renal inflammation and disease activity (20, 23, 171–174).

Neutrophils in SLE are hyperactivated, characterized by increased degranulation, elevated ROS production, and excessive NET formation. In active disease states, these cells spontaneously release NETs enriched with immunostimulatory components (e.g., LL-37/histone-DNA complexes), which activate pDCs and drive persistent type I interferon (particularly IFN-α) production. In turn, IFN-α promotes neutrophil activation via the JAK-STAT signaling pathway, establishing a pro-inflammatory positive feedback loop (24, 175, 176). Dysregulated NETosis is a hallmark of SLE: exaggerated NET release exposes nuclear autoantigens, which become targets of autoantibodies; concurrently, DNase I inhibitors and circulating anti-NET antibodies impair NET degradation, leading to sustained autoantigen exposure. Recent findings indicate that SLE neutrophils can undergo mitochondrial NETosis, releasing mitochondrial DNA (mtDNA), which contributes to elevated serum mtDNA levels observed in patients (177–180). Metabolic reprogramming further exacerbates neutrophil dysfunction in SLE, with increased glycolytic flux and mitochondrial impairment promoting ROS production and inflammatory cytokine release, thereby sustaining chronic inflammation (181, 182). Overactivation of the mTOR pathway drives metabolism toward glycolysis, while abnormal methylation of autophagy-related genes (such as ATG5) leads to delayed apoptosis and prolonged pro-injury activity of neutrophils (183). In addition, defective clearance of apoptotic neutrophils leads to accumulation of self-antigens, and impaired phagocytic function of macrophages and DCs further exacerbates immune activation (27).

The interaction between neutrophils and other immune cells is deeply involved in the immune imbalance of SLE: HMGB1 in NETs can enhance the antigen presentation ability of DCs; BAFF and APRIL secreted by neutrophils directly promote the differentiation of B cells into autoantibody-secreting plasma cells; the IL-6 and IL-23 released by them induce the polarization of Th17 cells, which recruit more neutrophils by secreting IL-17 to form a pro-inflammatory microenvironment (184). Ultimately, neutrophil dysregulation underpins multi-organ damage in SLE: infiltrating neutrophils release proteases, ROS, and NETs that drive glomerular inflammation in lupus nephritis; cutaneous lesions, vasculopathy, and pulmonary complications are likewise linked to neutrophil-mediated injury. Synergistic interactions with macrophages and DCs further intensify tissue destruction (173, 185, 186). Overall, neutrophils contribute to a self-perpetuating cycle of inflammation in SLE through aberrant NETosis, metabolic reprogramming (glycolysis/mTOR activation), and crosstalk with immune cells (pDCs, B cells, Th17), ultimately leading to autoantigen exposure, organ damage, and disease progression.

## 7 Dysfunctional NK cells and ILCs disrupt immune homeostasis and drive pathogenesis in SLE

### 7.1 NK cells

Natural killer (NK) cells are pivotal components of the innate immune system. Based on the expression level of CD56, NK cells are divided into two functional subgroups: the CD56^bright^ subgroup is mainly immune-regulated, regulating the activity of macrophages, DCs, and T/B cells through cytokines; the CD56^dim^ subgroup has stronger cytotoxicity and relies on granzyme B and perforin to directly kill target cells (187). Its functional balance is achieved by the dynamic regulation of activating receptors (such as NKG2D, NCR) and inhibitory receptors (such as KIR, NKG2A) - the former recognizes target cell stress ligands to trigger killing, and the latter binds to its own MHC class I molecules to prevent accidental injury to healthy tissues, jointly maintaining immune homeostasis (188).

**Figure 5:**
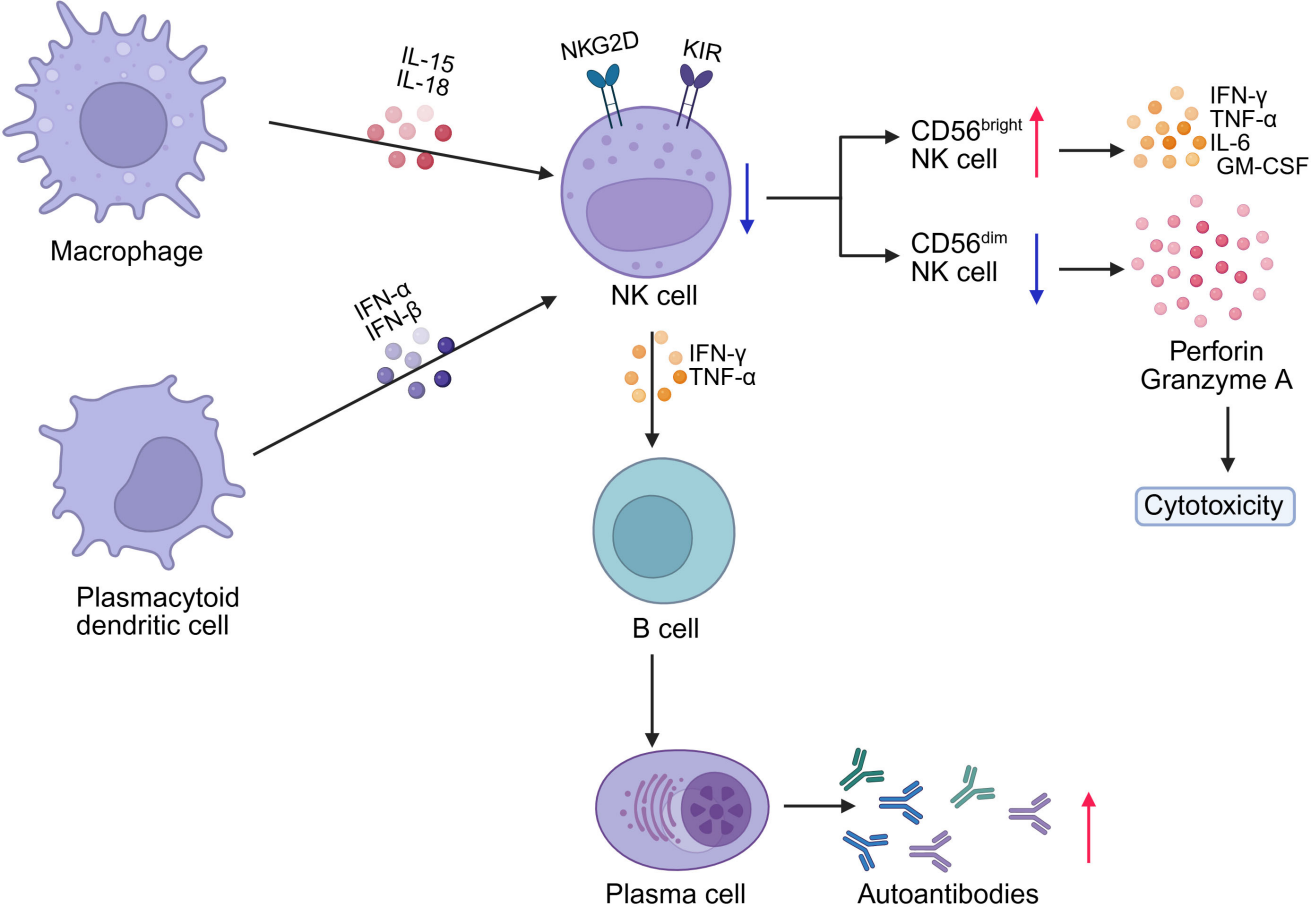
Dysregulation of NK cells and their pathogenic role in SLE. In SLE, NK cell function is impaired due to dysregulation of cytokine signals such as IL-15, IL-18 (from macrophages) and type I interferons (IFN-α, IFN-β from plasmacytoid dendritic cells). Activation receptors (NKG2D) and inhibitory receptors (KIR) on NK cells are imbalanced, leading to reduced cytotoxic capacity, diminished perforin and granzyme A secretion, and alterations in CD56^bright^ and CD56^dim^ NK cell subsets. Decreased NK cell-mediated cytotoxicity fails to clear autoreactive cells, while increased inflammatory cytokine production (IFN-γ, TNF-α, IL-6, GM-CSF) promotes chronic inflammation. Additionally, NK cells enhance B cell activation and plasma cell differentiation, further fueling the production of autoantibodies. This cascade contributes significantly to the inflammatory and autoimmune pathology observed in SLE.

In SLE, NK cell homeostasis is markedly perturbed (**Figure 5**). Active disease is characterized by a significant reduction in circulating NK cells—particularly within the CD56^dim^ cytotoxic subset—and impaired effector function. Downregulation of the activating receptor NKG2D and decreased secretion of granzyme B and perforin diminish the ability of NK cells to eliminate autoreactive B cells and apoptotic debris, thereby facilitating persistent autoantibody production (25, 26, 189). Moreover, aberrant expansion of the CD56^bright^ subset is observed, exhibiting altered functionality: while IFN-γ production is elevated, the release of immunomodulatory cytokines such as IL-6 and IL-10 is diminished, further promoting an inflammatory milieu and the activation of autoreactive T and B cells (178, 190). The intercellular interactions between NK cells and other immune components are also disrupted in SLE. Defective crosstalk with DCs impairs the clearance of apoptotic cells and enhances the presentation of self-antigens, while the inability of NK cells to regulate DC activity exacerbates autoimmunity (27, 191, 192). These multilayered dysfunctions contribute directly to organ pathology: in lupus nephritis, defective NK-mediated clearance of immune complexes aggravates renal inflammation; in the vascular system, NK cell dysfunction is implicated in endothelial damage and increased cardiovascular risk (28, 193, 194).

Emerging therapeutic strategies aim to restore NK cell functionality in SLE. These include cytokine-based interventions such as IL-15 and IFN-γ to enhance cytotoxicity, blockade of inhibitory receptors like NKG2A to release suppression of effector activity, and the use of hydroxychloroquine (HCQ) to correct mitochondrial dysfunction (195). These approaches offer promising avenues for reinstating NK cell-mediated immune regulation and ameliorating disease activity. In conclusion, NK cell dysfunction in SLE—characterized by diminished cytotoxicity, skewed cytokine profiles, and impaired immunoregulatory interactions—disrupts immune equilibrium, perpetuates inflammation, and drives tissue damage, ultimately contributing to disease progression.

### 7.2 ILCs

Innate lymphoid cells (ILCs) have emerged as key regulators of immune dysregulation in SLE. Flow cytometric analyses have revealed significant alterations in ILC homeostasis in SLE, with peripheral blood showing increased total Lin^-^CD127^+^CD45^+^ ILCs and selective expansion of pro-inflammatory ILC1 and ILC3 subsets, while ILC2 frequencies are reduced (196). This shift toward an inflammatory ILC profile correlates with disease activity and LN, suggesting that ILC imbalance may contribute to systemic inflammation and organ-specific pathology. IFN, a central cytokine in SLE pathogenesis, further shapes the ILC landscape. Patients exhibiting a strong type I IFN signature display reduced circulating ILC2 and ILC3 populations due to IFN-driven upregulation of Fas (CD95), a death receptor that promotes apoptosis of these subsets (197). This IFN-mediated depletion of ILC2s and ILC3s may reinforce the predominance of inflammatory ILC1s, amplifying immune dysregulation and contributing to chronic inflammation in SLE.

Beyond peripheral alterations, recent high-resolution tissue studies demonstrate that kidney-resident NKp46^+^ ILC1s act as potent amplifiers of LN. Rather than initiating autoimmune responses, these cells function downstream of autoantibody production to escalate local organ damage. Upon NKp46 engagement, ILC1s upregulate CSF2 (GM-CSF), promoting pathogenic monocyte-derived macrophage expansion and facilitating their infiltration into epithelial niches, thereby accelerating kidney injury. Targeting NKp46 signaling mitigates tissue damage, and similar NKp46–CSF2–macrophage interactions have been confirmed in human LN (198). Together, these findings identify ILCs—particularly tissue-resident NKp46^+^ ILC1s—as inflammatory rheostats in SLE, linking systemic cytokine imbalance with organ-specific pathology.

A comparative overview of the core mechanisms of immune dysregulation across major cell types in SLE is provided in **Table 5**.

**Table 5:** Comparative mechanisms of immune cell dysregulation in SLE.

Category	DCs	Macrophages	Neutrophils	NK Cells	References
Key Subsets	pDC, cDC1/cDC2	M1, M2	Low-density granulocytes (LDG)	CD56^bright^, CD56CD5^6dim^	(152, 199)
Core Dysregulation	pDC overproduction of IFN-α (TLR7/9) Increased cDC co-stimulation Loss of tolerance	M1/M2 polarization imbalance Defective efferocytosis Metabolic reprogramming (enhanced glycolysis/impaired OXPHOS)	Enhanced NETosis (TLR7/9 activation) Metabolic reprogramming (enhanced glycolysis/mTOR activation) Impaired apoptotic clearance	Reduced cytotoxicity (NKG2D downregulation) Cytokine imbalance (IFN-γ↓/IL-6↑) Immunoregulatory defects	(12, 13, 15, 152) (166, 172, 179) (25, 26)
Key Molecules/Pathways	TLR7/9, IFN-α, CD80/CD86, STAT1	NLRP3 inflammasome, iNOS, IL-6/TNF-α, MerTK	mTOR, NLRP3, HMGB1, BAFF/APRIL	NKG2D, KIR, granzyme B/perforin, IL-15 receptor	(11, 12, 18, 169) (25, 180, 184, 189)
Metabolic Features	–	Enhanced glycolysis, increased mitochondrial ROS, impaired OXPHOS	Enhanced glycolysis (mTOR activation), impaired mitophagy	Mitochondrial dysfunction (HCQ-reversible)	- (166, 169) (181, 182) (195)
Immune Interactions	Activation of autoreactive T/B cells Recruitment of monocytes/neutrophils	crosstalk with pDCs (IFN-α-driven) Promotion of Th17 differentiation	NET-mediated pDC activation (IFN-α loop) BAFF/APRIL-driven B cell activation	Defective DC crosstalk Impaired clearance of autoreactive B cells	(12, 13) (165, 170) (179, 184) (27, 191)
Epigenetic Abnormalities	–	Hypomethylation of pro-inflammatory genes (IL-6/TNF), upregulated miR-155	ATG5 hypermethylation	–	- (118) (176) -
Clearance Defects	Impaired apoptotic cell clearance	Reduced efferocytosis (MerTK downregulation)	Impaired NET clearance (DNase I inhibition)	Reduced target cell killing (granzyme B deficiency)	(13, 16, 151, 168) (178, 179) (25)
Target Organ Damage	Multi-organ immune complex deposition	Lupus nephritis (renal infiltration), skin/lung injury	Glomerular NET deposition, vasculitis	Renal/cardiovascular injury (impaired immune complex clearance)	(152, 157) (19, 164, 185) (173) (28, 193)
Therapeutic Targets	TLR7/9 inhibitors, IFN-α blockers	Polarization modulation (M2 induction), metabolic inhibitors (glycolysis)	NETosis inhibitors (PAD4 inhibitors), mTOR inhibitors	IL-15/IFN-γ therapy, NKG2A blockade, hydroxychloroquine	(12, 15, 158, 166) (23, 179) (195)

## 8 The gut-immune axis: microbiome dysbiosis in SLE pathogenesis

### 8.1 Overview of gut microbiome alterations in SLE

A growing body of research over the past decade has established gut microbiome dysbiosis as a key environmental factor contributing to immune dysregulation in SLE (200, 201). Alterations at the phyla level, including increased abundance of *Proteobacteria* and reduced *Firmicutes/Bacteroidetes* (F/B) ratio, have been consistently reported in SLE patients (202, 203). Reduced alpha diversity represents another reproducible finding across multiple independent cohorts (202, 204, 205). These compositional shifts have been proposed as signature markers of gut dysbiosis in SLE and may serve diagnostic potential. At the genus and species level, specific taxonomic alterations have been identified. SLE patients exhibit significant depletion of beneficial, butyrate-producing commensals, including *Faecalibacterium prausnitzii* and *Roseburia* (206, 207). *Faecalibacterium prausnitzii* exerts protective effects through multiple mechanisms, including short-chain fatty acid production, histone deacetylase inhibition, Treg cell induction, and enhancement of epithelial barrier integrity (208, 209). Conversely, enrichment of pro-inflammatory taxa including *Ruminococcus gnavus*, *Lactobacillus salivarius* and *Streptococcus anginosus* has been observed in SLE patients (204, 205, 210).

### 8.2 Mechanisms linking gut dysbiosis to SLE pathogenesis

The gut microbiome influences SLE pathogenesis through several interconnected mechanisms.

#### 8.2.1 Gut barrier dysfunction and microbial translocation

SLE patients frequently exhibit compromised intestinal barrier integrity (211). Microbial translocation through a disrupted intestinal barrier represents a key mechanism linking dysbiosis to systemic autoimmunity (212). Upon breaching the gut epithelial barrier, translocated commensals such as *Enterococcus gallinarum* and *Ruminococcus gnavus* can disseminate to extraintestinal sites including mesenteric lymph nodes, liver, and spleen, where they activate autoreactive T and B cells through mechanisms involving molecular mimicry and Th17 polarization, ultimately driving the production of autoantibodies and end-organ damage in lupus (213–215). Meanwhile, disruption of tight junction proteins allows bacterial products—including lipopolysaccharide and unmethylated CpG DNA—to translocate into the systemic circulation, where they activate Toll-like receptor 9 (TLR9) on circulating immune cells, amplifying systemic inflammation, type I interferon production, and autoantibody generation (216, 217).

#### 8.2.2 Molecular mimicry and cross-reactivity

Microbial antigens sharing sequence or structural homology with self-antigens may trigger autoreactive B and T cells through molecular mimicry (218, 219). *Ruminococcus gnavus* has emerged as a particularly important pathobiont in SLE, especially in lupus nephritis. Patients with flares of lupus nephritis and intestinal expansions of *Ruminococcus gnavus* display whole blood transcriptome profiles indicative of platelet, neutrophil, and myeloid cell activation. Serum levels of Platelet Factor 4 and neutrophil extracellular traps are significantly elevated in these patients and correlate with levels of immunoglobulin G antibodies to a novel lipoglycan produced by pathogenic *Ruminococcus gnavus* strains (205, 220). *Bacteroides thetaiotaomicron* has also been specifically implicated in lupus nephritis pathogenesis (221, 222).

#### 8.2.3 Microbial metabolites and immune modulation

SCFAs, including butyrate, propionate, and acetate, are produced by commensal bacteria through dietary fiber fermentation. These metabolites play critical roles in maintaining gut barrier integrity and regulating immune responses (223). SCFAs promote Treg cell differentiation and interleukin-10 production via GPR41/GPR43 signaling and histone deacetylase inhibition (224). Butyrate functions as a primary energy source for colonocytes while simultaneously modulating host immunity through GPR43-mediated signaling and epigenetic regulation (225). In SLE, levels of bacterial metabolites such as butyrate and other SCFAs appear to play a key role in modulating disease activity (226, 227). Dysbiosis promotes alterations in IFN-γ levels and the balance between Treg cells and Th17 subsets, with SCFA deficiency contributing to impaired Treg cell function and heightened Th17 responses (31, 228).

### 8.3 Clinical correlations and treatment effects

Multiple studies have demonstrated associations between specific microbial alterations and clinical parameters in SLE (200, 229). Reduced microbial diversity and decreased *Faecalibacterium prausnitzii* abundance inversely correlate with SLEDAI scores (230). Enrichment of *Ruminococcus gnavus* and *Bacteroides thetaiotaomicron* has been specifically associated with renal involvement in lupus nephritis. Notably, SLE medications influence the gut microbiome. Patients receiving mycophenolate mofetil display a significantly higher Firmicutes/Bacteroidetes ratio than non-users, suggesting that mycophenolate mofetil may restore Firmicutes colonization and enhance butyrate levels (31, 231).

### 8.4 Therapeutic implications of microbiome modulation

The recognition of gut dysbiosis as a pathogenic factor in SLE has opened new therapeutic avenues. Microbiota-based therapy appears promising and includes dietary interventions, prebiotics, probiotics, symbiotics, and fecal microbiota transplantation (FMT) (5, 31, 200). FMT demonstrates promising therapeutic potential by restoring microbial balance, enhancing immune regulation, and improving metabolic homeostasis (232, 233). Probiotic supplementation with Lactobacillus and Bifidobacterium strains has shown potential in maintaining intestinal barrier integrity and immune function (234). Dietary interventions such as high-fiber diets increase SCFA production, which modulates inflammation and supports gut health. Postbiotic metabolites, particularly direct administration of butyrate or other short-chain fatty acids, represent a promising approach to bypass the complexity of live bacteria while achieving immune-modulatory effects (228, 235).

## 9 Conclusion and outlook

**Figure 6:**
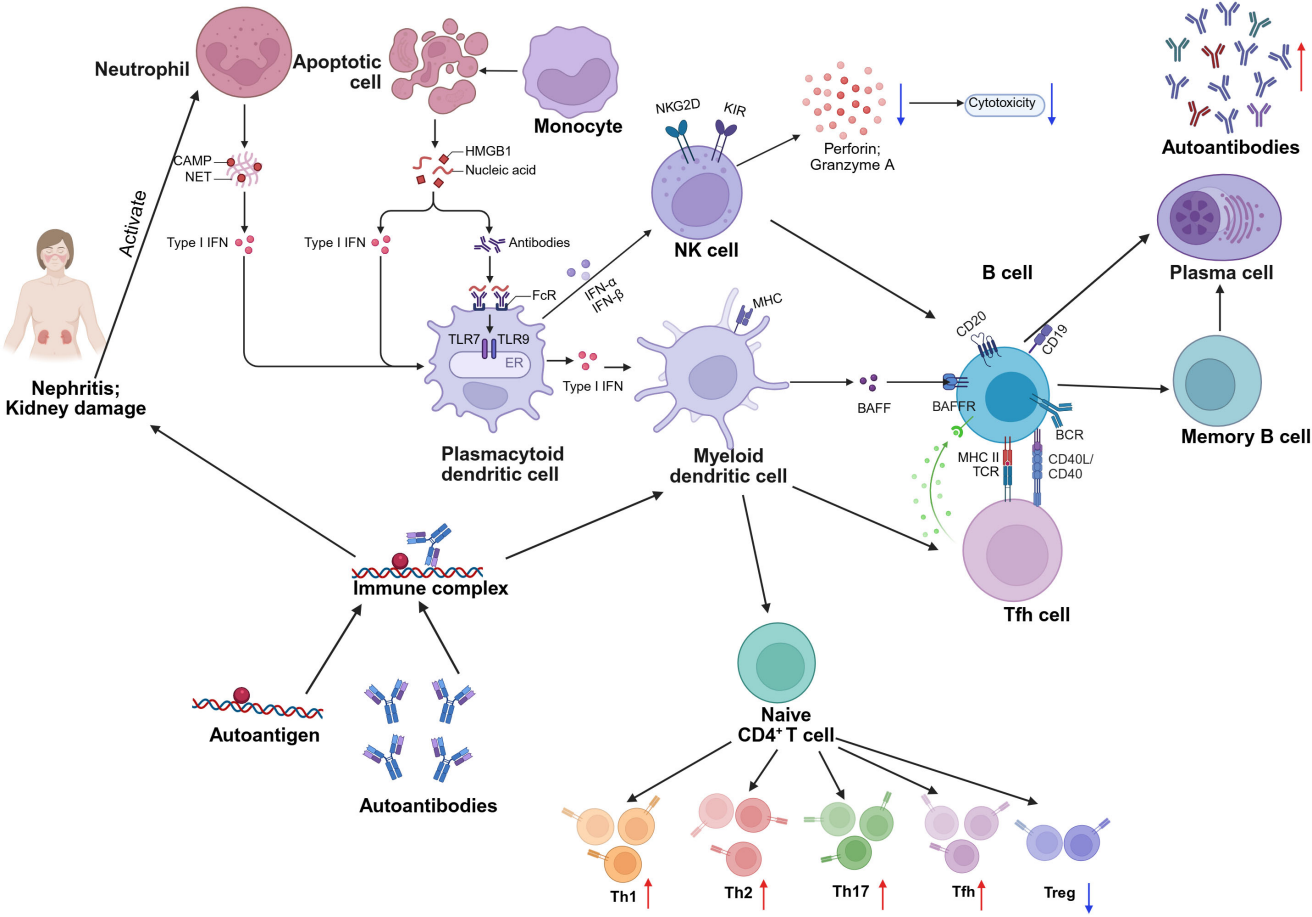
Immune cell dysregulation in the pathogenesis of SLE. This figure shows the complex network of immune cell interactions that contribute to the development and progression of SLE. Aberrant clearance of apoptotic cells and activation of neutrophils promote the release of nucleic acids and type I interferons, which promote activation of pDCs through TLR7/9 signaling. In turn, pDCs secrete additional type I interferons, further activating NK cells, myeloid dendritic cells, and B cells. NK cells in SLE exhibit reduced cytotoxicity but promote inflammation and B cell activation. Overproduction of BAFF by myeloid dendritic cells enhances B cell survival, differentiation into plasma cells, and production of autoantibodies. Tfh provide additional support for B cell maturation. At the same time, naive CD4^+^ T cells differentiate into inflammatory Th1, Th2, Th17, and Tfh subsets, and Treg function is impaired, promoting autoimmunity. Immune complex deposition can lead to tissue damage, including nephritis and kidney damage, which is one of the hallmark features of severe SLE.

The pathogenesis of SLE is driven by multifaceted dysregulation across both adaptive and innate immunity, with aberrant T and B cell activation playing a central role (**Figure 6**). Expansion of Th17 and Tfh subsets, coupled with impaired Treg function, promotes sustained inflammation and high-affinity autoantibody production, ultimately leading to immune complex deposition and tissue damage (59, 236). T cell metabolic abnormalities—such as elevated glycolysis and mitochondrial dysfunction—not only sustain pathological activation but also provide actionable targets for metabolic intervention (e.g., mTOR inhibitors, glycolysis blockers) (41). Additionally, T and B cells form a pathogenic feedback loop via CD40–CD40L and IL-21 signaling, exacerbated by deficient Breg-mediated regulation, underscoring the need to restore immune tolerance (237). Innate immune dysregulation further amplifies disease. Persistent IFN-α production by pDCs via TLR7/9, M1-biased macrophage polarization, defective apoptotic clearance, and mitochondrial ROS accumulation collectively fuel chronic inflammation (11, 15). Excessive NETosis and LDG accumulation expose nuclear autoantigens, augmenting autoantibody responses and damaging endothelium, particularly in lupus nephritis (20). Impaired NK cytotoxicity and cytokine imbalance hinder clearance of autoreactive cells, destabilizing immune homeostasis (25). Emerging evidence has established gut microbiome dysbiosis as a key environmental factor contributing to immune dysregulation in SLE. Gut microbiome dysbiosis, characterized by depletion of butyrate-producing bacteria and enrichment of pro-inflammatory taxa such as *Ruminococcus gnavus*, further contributes to immune dysregulation through gut barrier dysfunction, molecular mimicry, and SCFA deficiency (200, 238).

Current treatment of SLE follows a treat-to-target strategy, combining universal background therapy with hydroxychloroquine, judicious glucocorticoid use, and steroid-sparing immunosuppressants such as mycophenolate mofetil, azathioprine, and cyclophosphamide, selected according to disease severity and organ involvement (239). Among these, hydroxychloroquine remains foundational due to its ability to reduce disease activity, prevent flares, and provide long-term vascular protection (240). The growing understanding of SLE pathogenesis has guided the development of targeted biologics. Given the central role of B cell hyperactivity driven by BLyS overexpression, belimumab (anti-BLyS) has demonstrated efficacy in systemic disease and renal subsets (241). Similarly, recognizing that persistent type I interferon production by pDCs amplifies inflammation, anifrolumab (IFN-I receptor blockade) increases the probability of achieving low disease activity or remission in randomized clinical trials (241). For LN, the calcineurin inhibitor voclosporin, when combined with standard induction therapy, provides higher complete renal response rates and is now an approved kidney-specific therapy (242). For patients with treatment-refractory SLE, emerging cellular immunotherapies are redefining therapeutic expectations by targeting the pathogenic B-cell and plasma-cell compartments. Novel chimeric antigen receptor (CAR) approaches directed against CD19 have shown early promise, and dual CD19/BCMA targeting further addresses persistent autoreactivity driven by CD19^+^ B cells and long-lived CD19^-^BCMA^+^ plasma cells (243). In a phase 1 trial (NCT05030779), co-infused CD19- and BCMA-CAR T cells achieved rapid clinical remission, with 80% of patients attaining both DORIS remission and LLDAS by week 12, alongside eradication of autoreactive clones and restoration of immune homeostasis, suggesting durable or potentially curative benefit (244). Beyond autologous CAR-T approaches, allogeneic CAR-engineered NK-cell therapy offers a scalable and potentially safer alternative. In a first-in-human study (NCT06010472), CAR-NK therapy demonstrated favorable safety, with only mild cytokine release syndrome and no neurotoxicity, while achieving DORIS remission or low disease activity in 67% of patients with ≥12-month follow-up. As an off-the-shelf cellular product, CAR-NK therapy may overcome the manufacturing delays, cost barriers, and infection risks associated with autologous CAR-T therapy, positioning it as a next-generation option for severe and refractory disease (245). Microbiome-based therapies, including fecal microbiota transplantation and probiotic supplementation, represent emerging complementary strategies to restore immune balance (232, 246). Overall, the evolution of SLE therapy now spans from conventional immunomodulation to precision biologics and advanced cellular strategies, with the latter offering the potential to eliminate pathogenic immune memory rather than merely suppress ongoing inflammation.

In summary, deciphering the heterogeneity of SLE and developing stratified, precision-based therapies remain crucial. Integration of biomarker-driven classification, immune metabolic profiling, and tissue-specific immune targeting may enable the shift from symptom control toward durable remission or even a functional cure.
